# Highly Effective Fibrinolysis by a Sequential Synergistic Combination of Mini-Dose tPA plus Low-Dose Mutant proUK

**DOI:** 10.1371/journal.pone.0122018

**Published:** 2015-03-26

**Authors:** Ralph Pannell, Shelley Li, Victor Gurewich

**Affiliations:** Vascular Research Laboratory, Mount Auburn Hospital, Harvard Medical School, Cambridge, Massachusetts, United States of America; McGill University Health Center / Royal Victoria, CANADA

## Abstract

Results of thrombolysis by monotherapy with either tPA or proUK have not lived up to expectations. Since these natural activators are inherently complementary, this property can be utilized to a synergistic advantage; and yet, this has undergone little evaluation. ProUK is no longer available because at pharmacological concentrations it converts to UK in plasma. Therefore, a single site proUK mutant, M5, was developed to address this problem and was used in this study. Fibrinolysis was measured using preformed fluoresceinated 24 h old clots in a plasma milieu rather than by the standard automated method, because proUK/M5 is sensitive to inactivation by thrombin and activation by plasmin. The shortest 50% clot lysis time that could be achieved by tPA or M5 alone was determined: mean times were 55 and 48 minutes respectively. These bench marks were matched by 6% of the tPA monotherapy dose combined with 40% that of M5: mean lysis time 47 minutes with less associated fibrinogenolysis. Results showed that the tPA effect was limited to initiating fibrinolysis which was completed by M5 and then tcM5. Plasma C1-inhibitor inhibited fibrinogenolysis by M5, providing protection from side effects not available for proUK. In conclusion, by utilizing the complementary properties and sequential modes of action of each activator, more efficient fibrinolysis with less non-specific effects can be achieved than with traditional monotherapy. *In vivo* validation is needed, but in a previous clinical trial using a similar combination of tPA and proUK (5% and 50% monotherapy doses) very promising results have already been obtained.

## Introduction

Thrombolysis with tPA is the current standard for this therapeutic modality. After important successes in acute myocardial infarction (AMI), tPA has since been replaced by percutaneous coronary intervention (PCI) as the treatment of choice, despite PCI being much more time-consuming and costly. Two problems may have contributed to this outcome. First, tPA’s therapeutic efficacy was compromised by a dose-limiting incidence of intracranial hemorrhage (ICH) [1]. Second, there was a relatively high coronary rethrombosis rate with tPA [2, 3] associated with hematological evidence of thrombin generation [4–6].

In ischemic stroke, a further dose reduction was required due to a 20% incidence of ICH when tPA doses equivalent to those in AMI were used [7], and reocclusion rates of 14–31% were reported [8–10]. In a national estimate of use, only 2–5% of patients with ischemic stroke were treated with tPA in the US [11], though the percentage is higher in dedicated stroke centers.

These limitations of therapeutic thrombolysis reflect the experience with tPA thrombolytic monotherapy. In contrast to monotherapy, certain previous studies showed that tPA may be more effective when combined with the other natural plasminogen activator, prourokinase (proUK), due to a synergistic combined effect [12, 13], although others did not find this synergism [14]. The mechanism responsible for synergy has also been described [15], but proUK is no longer available. The proUK mutant, M5, has the same mechanism of action so should also be synergistic with tPA, but this has not, heretofore, been established.

Certain potentially useful clinical properties of proUK were established during its clinical trials, including phase III trials in AMI [16–18]. For example, proUK induced little (5%) [17] or no [19] rethrombosis and did not induce a procoagulant effect in blood [19] in contrast to tPA. Unfortunately, at therapeutic concentrations in plasma, the intrinsic activity of proUK [20] was sufficient to induce systemic plasmin generation, resulting in systemic conversion of proUK to urokinase (UK) and consequent loss of fibrin-specificity. As a result of the bleeding hazard from this, marketing approval for proUK was denied by the European Medicines Agency (EMEA). In order to address this problem, a more stable mutant proUK, designated M5, was developed and was used in the present study.

M5 is a single site mutant (Lysine 300→Histidine) of proUK with a lower single-chain intrinsic activity [21] but with the same two-chain M5 (tcM5) activity as UK [22], and its fibrinolytic mode of action did not differ from that of proUK. In a thromboembolic model in dogs, M5 thrombolysis was associated with significantly less bleeding than that by native proUK or tPA [23]. This was found to be due to an unusual inhibition of tcM5 by C1-inhibitor in plasma [24], an effect that could be augmented by adding pharmaceutical C1-inhibitor which inhibited fibrinogenolysis but not fibrinolysis by M5 [25, 26].

In the present study, the fibrinolytic and fibrinogenolytic effects of tPA or M5 alone, at doses which induced the shortest lysis time achievable, were compared with those of fractional combinations. Standardized clots in a plasma milieu were used in the fibrinolytic assay, since the standard, automated assay was precluded for use with proUK/M5 due to its sensitivity to thrombin and plasmin, which respectively inactivate and activate the proenzyme. The findings showed that a synergistic fractional dose combination induced optimal lysis times with fewer non-specific effects, which could be further attenuated by C1-inhibitor. The results suggest that combination thrombolytic therapy is more effective, safer, and more rational than monotherapy with either activator alone.

## Methods and Materials

### Materials

Mutant proUK (M5) from *E coli* was prepared by PxTherapeutics (Grenoble, France). Single-chain tissue plasminogen activator (tPA) (CathFlo) was obtained from Genentech (South San Francisco, CA). Fibrinogen was human, Kabi Grade L (Chromogenix, Milan, Italy). Aprotinin and fluorescein isothiocyanate were from Sigma Chemicals, St. Louis, MO. Thrombin (ThromboMax, 100 NIH U per ml) was obtained from Sigma (St Louis, MO). C1-inhibitor was clinical grade from CSL Behring, Marburg, Germany. Human outdated bank plasma, pooled from four donors, was used.

### Methods

#### Explanation of Methodology.

Studies of fibrinolysis by proUK cannot use the automated clotting/lysis assay which has become standard for other activators. When this is used with proUK, thrombin inactivates some of the proUK at the outset, and whatever proUK remains will then be converted quickly to UK during the lytic phase by plasmin. As a result, the standard assay measures the fibrinolytic activity of the remaining UK, a non-specific activator, but not that of proUK. Therefore, a 24 h old preformed clot was used in a plasma milieu. A plasma milieu is essential, not only to approximate *in vivo* lysis, but because in the absence of inhibitors, the intrinsic activity of proUK/M5 activates plasminogen and plasmin then converts proUK/M5 to UK/tcM5.

In previous studies of fibrinolysis with proUK using this clot lysis model, the results obtained correlated well with those later found in clinical studies. For example, conversion of proUK to UK above a certain concentration in plasma was seen during clot lysis *in vitro* and in the clinical trials; synergy was identified first in this model *in vitro* and then found *in vivo*; and even the plasma concentration of proUK required for effective clot lysis in this model (1.5 μg/ml) and that used clinically (2–3 μg/ml steady state concentration) were similar. Since proUK is a very species-specific activator, the *in vitro* results in human plasma often correlated better with clinical findings than those from animal models.

#### Clot Lysis Experiments.

Clots were made up from 0.2 ml pooled blood bank plasma by recalcification (35 mM) with the addition of a trace of thromboplastin and a tracer of fluoresceinated fibrinogen [27]. The clots were first incubated at 37°C for 1 hour and then overnight at room temperature. The following day, the clots were placed into 2 ml of pooled bank plasma and the activators added as specified. Clot lysis was monitored by taking 50 μl samples at time points for reading of fluorescence, representing fibrin degradation products released from the clots.

The lysis curves were plotted as percent lysis over time. The 100% point was obtained from the mean of the highest readings at complete lysis. The base line reading was obtained at onset, since the plasma shows fluorescence (~15% of the full signal) which was subtracted to obtain the zero point. The time to 50% lysis under each of the experimental conditions was determined from the lysis graphs and was used as the principal endpoint.

Each clot lysis experiment was done in triplicate. Graph Pad Prism was used for preparing graphs and for statistical analysis (means and Standard Deviation).

#### Fibrinogen determination.

After clot lysis had gone to completion, a final plasma sample (1.0 ml) was obtained for determination of fibrinogen. Further proteolysis was prevented by the addition of aprotinin (200 KIU/ml) to the sample. The results were recorded as percent of the baseline (BL) fibrinogen obtained from a plasma sample before addition of the activators.

Fibrinogen was measured as thrombin-clottable protein. After dilution of the plasma sample with 1 vol. phosphate-buffered saline, thrombin (200 μl of 1,000 NIH units/ml) was added, mixed gently, and incubated for 1 hour at 37°C, and then overnight at room temp. The following morning, each clot was wound onto a thin, long stemmed plastic transfer pipette tip to which the gel adhered, and the serum content was expressed by pressure against the test tube wall and then against a paper towel. The white-appearing fibrin on the pipette stem was then placed in >5ml saline for >1h to allow diffusion of any remaining serum proteins. Then the fibrin was peeled off the tip and placed into 1 ml of 5% NaOH, boiled for 1 min, and then kept at room temp until all fibrin had gone into solution. The protein in the solution was measured spectrophotometrically at 280 nm.

#### Experimental Plan and Conditions.

The shortest time to lysis obtainable by the activators alone and the fibrinogen that remained at the end of lysis were first determined. This was done by incremental increases of dosing of the activators until no further shortening of the lysis time occurred. This lysis time served as the benchmark against which various combinations of tPA and M5 were compared, in order to select the combination which induced a comparable effect. Once a combination was selected, the contributions to lysis of the components separately were determined from the results. Evaluation of the effect of C1-inhibitor on fibrinolysis and fibrinogenolysis was also determined.

a) *Determination of the shortest time to lysis and fibrinogenolysis for tPA and M5 alone*: The shortest time was defined as that at which no further dose-dependent shortening of the lysis time could be induced. The corresponding dose was called the maximum dose. The time to 50% clot lysis was determined from the lysis curves for tPA and M5.

b) *The effect of C1-inhibitor on fibrinogenolysis*: C1-inhibitor (750 μg/ml) was added to the plasma prior to clot lysis by M5 at the three highest doses. In studies with the low-dose combination, the C1-inhibitor was added 30 minutes after the activators, since previous studies showed that C1-inhibitor inhibits tPA-mediated fibrinolysis [28].

c) *Determining a fractional combination of tPA and M5 that induces a time to 50% lysis comparabl*e *to maximum-dose monotherapy*: Lysis by numerous combinations and ratios of tPA and M5 were tested. The criterion for selection was the lowest dose of each activator that in combination with the other induced a 50% lysis time comparable to the monotherapy benchmark. Fibrinogen remaining in plasma was determined at the end of lysis.

In some experiments, the effect of C1-inhibitor (750 μg/ml) on fibrinogenolysis and fibrinolysis was tested.

d) *The effect on lysis of a range of doses of tPA in combination with selected M5 dose in the fractional combination*: The effect of increasing tPA doses from 0.2 to 3 μg/ml (the maximum dose) in combination with M5 (6 μg/ml) was tested. The purpose of this experiment was to determine if tPA had a lytic effect beyond that of the lowest dose which was required for the initiation of fibrinolysis.

e) The mean lysis times and SD obtained in all experiments under the different conditions were calculated and presented together in a bar graph.

f) The hematological effect of the synergistic combination of tPA and M5 when incubated (37°C) in plasma alone without a clot was determined. Plasma samples were obtained hourly for fibrinogen determination over a 5h period.

## Results

### Shortest 50% clot lysis time achievable by tPA or M5 alone

**Fig 1:**
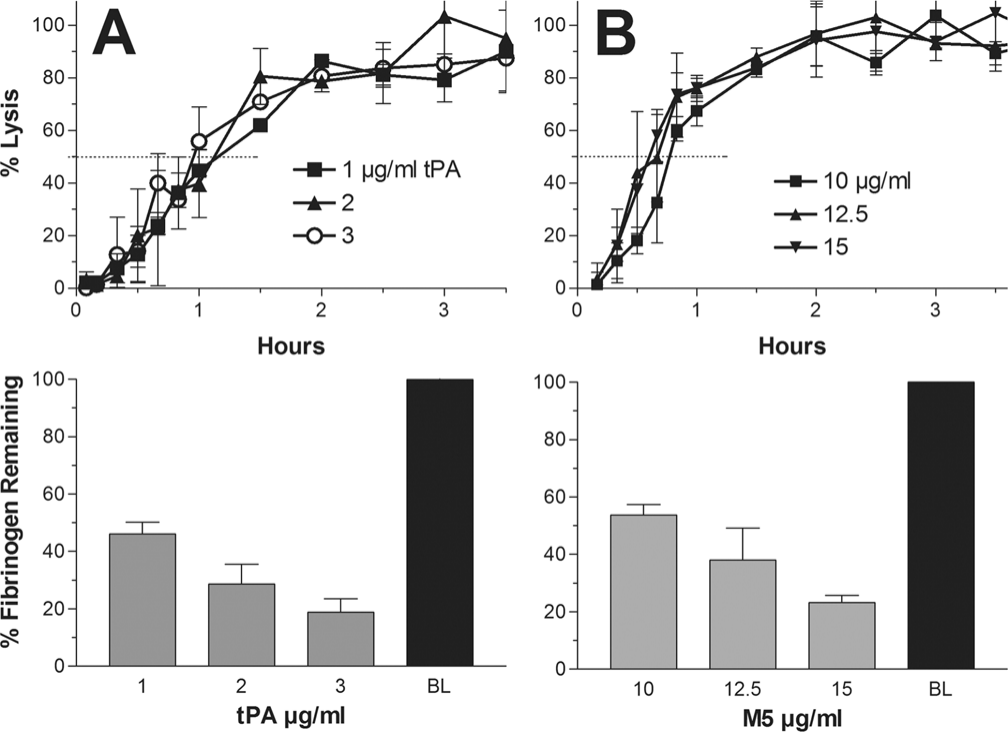
Maximum dose lysis: Representative experiments showing clot lysis with fluorescein-labeled plasma clots by tPA (A) and M5 (B) using the three highest doses up to the point at which no further shortening of the lysis time could be achieved. This represented the shortest lysis time achievable by each activator alone, which was induced by 3 μg/ml tPA or 15 μg/ml M5. In the representative experiment shown, the shortest time to 50% lysis was 60 minutes for tPA and 50 minutes for M5. In the bar graphs below, the fibrinogen concentrations at the end of clot lysis are shown, expressed as per cent of baseline (BL). As much 80% of fibrinogen was lost to degradation.

For tPA, an upper dose range of 1–3 μg/ml induced near to maximal lysis, and 3 μg/ml was the maximum dose beyond which no further shortening of the lysis time was inducible. A representative experiment with the top three doses is shown in which a 50% lysis time of 60 minutes was found. The fibrinogen concentration at the end of lysis at each of these doses, expressed as a percent of baseline, ranged from 19–45% of baseline and is shown in the bar graph below, mean and SD values (Fig. 1A).

For M5, an upper dose range of 10–15 μg/ml induced near to maximal lysis, and 15 μg/ml was the dose at which no further shortening of the lysis time was seen. A representative experiment is shown in which the 50% lysis time was 50 minutes. The corresponding fibrinogen concentration at the end of lysis at the three doses ranged from 25–55% of baseline and is shown in the bar graph below (Fig. 1B).

### Effect of C1-inhibitor on fibrinogenolysis by M5

**Fig 2:**
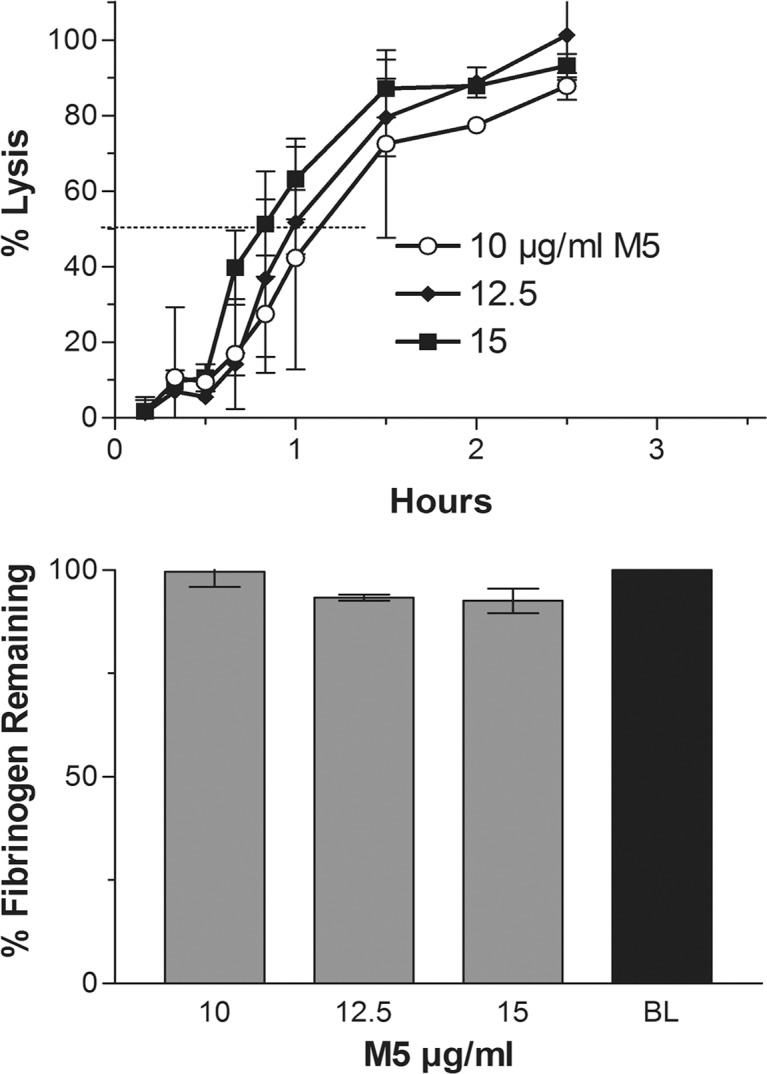
Effect of C1-inibitor: The experiment shown in Fig. 1B with M5 was repeated with prior addition of C1-inhibitor (750 μg/ml) to the plasma. The lysis rate was not inhibited, in fact was shortened, fibrinogenolysis was prevented. The experiment was not repeated with tPA because C1-inhibitor inhibits lysis by tPA (28) and seen also in Fig. 3B.

The experiment with the highest doses of M5 (10–15 μg/ml) was repeated in the presence of supplemental C1-inhibitor (750 μg/ml) added to the plasma before the addition of M5. As shown in Fig. 2 (top) this had no effect on the 50% clot lysis time which was 50 minutes. However, as shown in the bar graph (below), fibrinogen degradation was essentially prevented (1B). The inhibition of M5-induced fibrinogenolysis but not fibrinolysis by this inhibitor was previously reported [25, 26].

**Fig 3:**
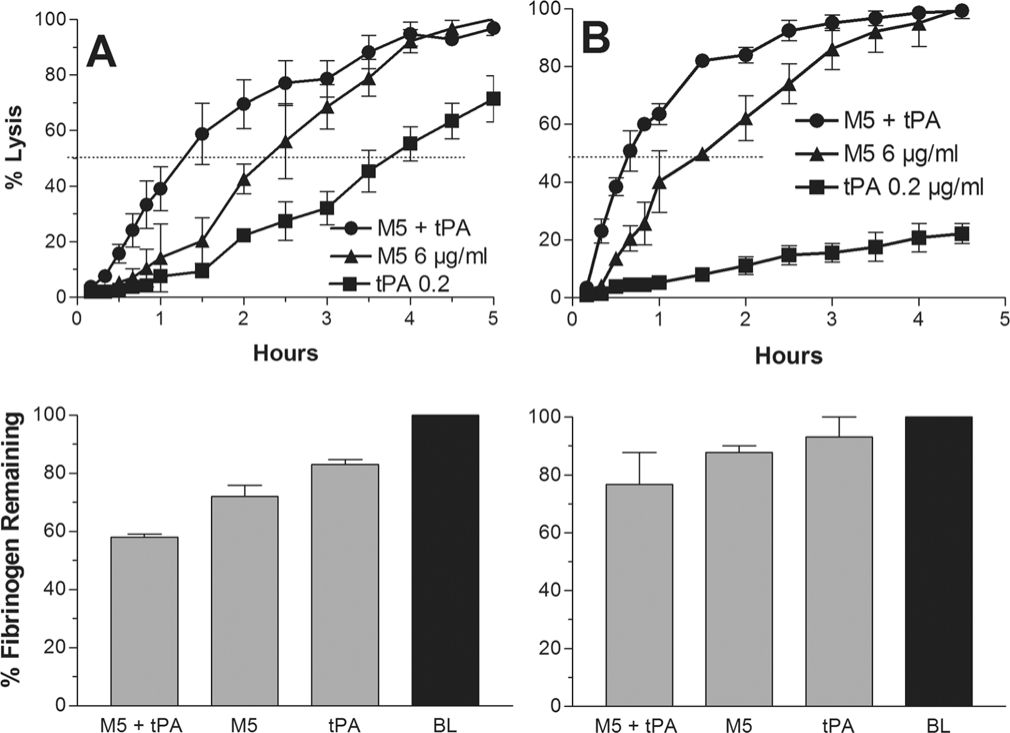
The synergistic combination: (A) Representative clot lysis experiment using the synergistic combination (0.2 μg/ml tPA + 6 μg/ml M5) (circle), which induced a 50% lysis time of 75 minutes. At this dose of M5 alone (triangle), the lysis time was 135 minutes, and tPA alone (square) induced a lysis time of 225 minutes. The findings show that a mini-dose tPA caused about a 45% shortening of the onset of lysis by M5. The plasma fibrinogens at the end of lysis expressed as %BL fibrinogen are shown below. (B) The same experiment as above except that C1-inhibitor (750 μg/ml) was added 30 minutes after the addition of the activators. As shown, this did not inhibit lysis, which was shortened to 30 minute, but did inhibit lysis by tPA alone. The C1-inhibitor attenuated fibrinogenolysis, as shown below.

The C1-inhibitor experiment was not repeated with tPA, since the C1-inhibition of fibrinogenolysis by tPA was previously shown to be accompanied by comparable inhibition of fibrinolysis (28) as also seen in Fig. 3B (top) versus 3A (top).

### Identification of a synergistic combination of tPA and M5

After multiple dose combinations of tPA and M5 were tested, the lowest dose of each activator was chosen (0.2 μg/ml tPA and 6 μg/ml M5) which when used in combination induced a lysis time equivalent to the shortest achievable with monotherapy. In some experiments even 0.1 μg/ml of tPA was sufficient to achieve this lysis time in combination with M5. These were fractional concentrations consisting of 6% of the tPA and 40% of the M5 monotherapy dose needed to induce a comparable lysis time, consistent with a synergistic effect. In the representative experiment shown, the 50% lysis time for the combination was 75 minutes, whereas it was 135 minutes for M5 (6 μg/ml) alone, indicating about a 45% shortening of the M5 lysis time by the small dose of tPA. The lysis time by tPA alone at this dose (0.2 μg/ml) was 225 minutes (Fig. 3A).

When the experiment was repeated with C1-inhibitor added 30 minutes after onset, fibrinogenolysis was inhibited but not fibrinolysis by the combination. In fact the lysis time was shortened to 30 minutes, an effect that may have been related to conservation of M5 and plasminogen by the inhibitor. By contrast, fibrinolysis by the tPA alone was inhibited, as previously also found *in vivo* [28] (Fig. 3B).

### Clot lysis by a fixed dose of M5 in combination with a range of tPA doses

**Fig 4:**
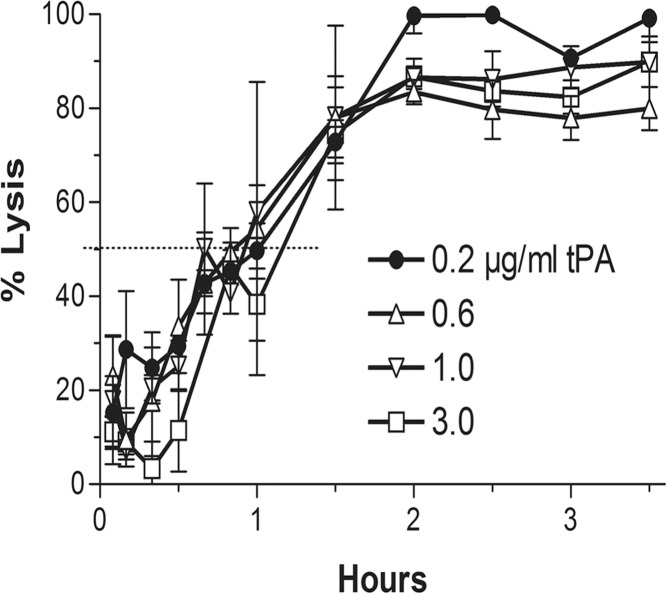
Lytic effect of range of tPA doses in presence of fixed low-dose M5: A representative experiment in which clot lysis by the synergistic dose of M5 (6μg/ml) was combined with a range of doses of tPA, from the synergistic dose (0.2 μg/ml) to the maximum lysis dose (3 μg/ml). No tPA lytic effect was detected beyond that obtained with the lowest dose. The findings were consistent with the tPA lytic effect being confined to the initiation of clot lysis.

The finding that tPA induced a major shortening of the M5 lysis time (Fig. 3) was consistent with its function to initiate fibrinolysis by activating plasminogen on intact fibrin [33,34]. In order to determine if tPA induced any additional lytic effect in presence of M5, a range of tPA doses (0.2–3 μg/ml) was added to the fixed M5 dose of the combination (6 μg/ml). A 50% lysis time of 48–60 minutes was found at all tPA doses without a discernible lytic effect beyond that obtained with 0.2 μg/ml, consistent with its lytic effect being limited to that of the initiation of fibrinolysis (Fig. 4).

### Mean and range of all the 50% clot lysis times

**Fig 5:**
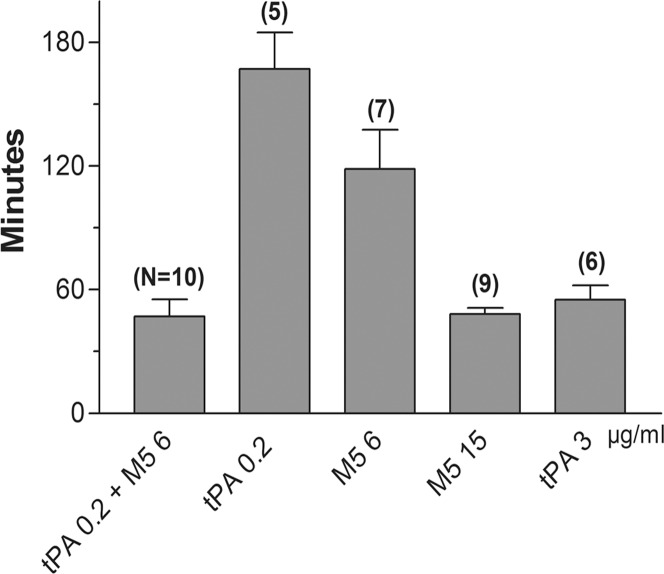
Lysis times from all the groups tested: The 50% lysis times (mean values and SD) from all the experiments in the different groups: synergistic combination (47 ± 2.5); the low doses of tPA (156 ± 5.3) and M5 (118 ± 7.2) in the synergistic combination when used alone; and the maximum doses of M5 (48 ± 1.6) and tPA (55 ± 1.5). The number of experiments is shown in brackets above each bar; the numbers below the bars are the μg/ml.

The minutes (mean and SD) of the 50% clot lysis times from all the experiments under the five different conditions are shown. The number of experiments is in brackets. The synergistic combination of 0.2 μg/ml tPA plus 6 μg/ml M5 induced an overall mean lysis rate of 47 [± 2.5] minutes; 0.2 μg/ml tPA alone was 156 [± 5.3] minutes; 6 μg/ml of M5 alone was 118 [± 7.2] minutes; 15 μg/ml of M5 alone was 48 [± 1.6] minutes; and 3 μg/ml of tPA alone was 55 [± 5] minutes. The shortest lysis time by the synergistic combination was lower than that by maximum dose tPA, but this difference did not reach significance (Fig. 5).

### Fibrinogenolysis by the synergistic combination in the absence of a clot

**Fig 6:**
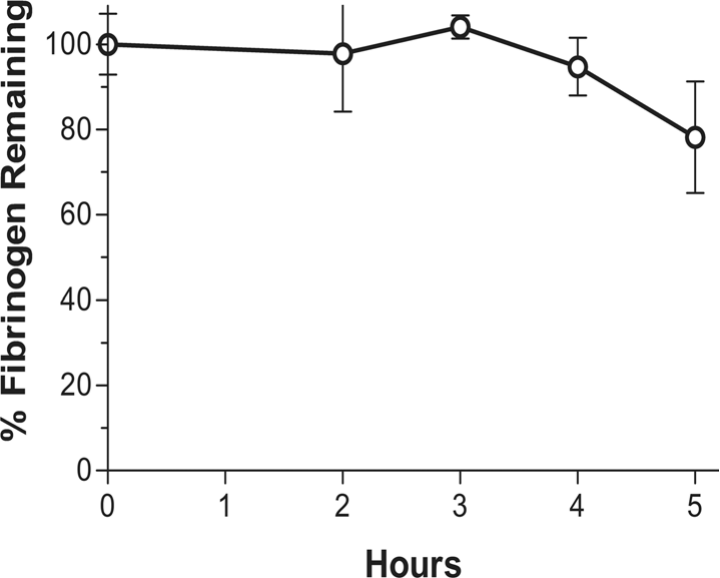
The effect of the synergistic dose combination in plasma alone: The synergistic combination of the activators (0.2 μg/ml tPA and 6 μg/ml M5) was incubated (37°C) in plasma without a clot. No fibrinogenolysis occurred for at least 3 h, showing that the plasminogen activation seen with the synergistic combination was fibrin-dependent and no non-specific effect was detected by the synergistic combination.

Fibrinogen degradation in plasma by the synergistic combination (0.2 μg/ml tPA + 6 μg/ml M5) alone over 2–5 h is shown (Fig. 6). There was no fibrinogen degradation seen for at least 3 h, which is a period well beyond the duration of therapeutic thrombolysis for stroke or AMI. This finding also indicates that the plasminogen activation which was seen in the lysis experiments by the combination was limited to that which was fibrin-dependent.

## Discussion

There are two plasminogen activators in human plasma, tPA and proUK. They share a common substrate but are otherwise notably different in enzymatic nature, fibrin affinity, and mode of action. Although therapeutic thrombolysis has invariably been by monotherapy, most often with tPA, the natural system employs both activators to a synergistic advantage. This biological paradigm was investigated using a single-site mutant of proUK (designated M5) in place of native proUK in a standardized model of clot lysis in a plasma milieu.

The shortest time to 50% clot lysis achievable by tPA or M5 alone was first determined and became the benchmark against which the efficacy of different combinations of the activators was compared. The respective shortest mean lysis times for tPA and M5 alone were 55 and 48 minutes and required 3 μg/ml and 15 μg/ml respectively. Extensive fibrinogenolysis accompanied these doses (Fig. 1A & B), but with M5 this could be prevented by supplemental C1-inhibitor without interfering with the rate of clot lysis (Fig. 2), whereas C1-inhibitor inhibited fibrinolysis by tPA (Fig. 3B), as also found previously in an animal model [28]. These optimally lytic doses of tPA are, of course, precluded clinically by ICH (1), especially in stroke where a further dose reduction is needed [7].

After testing multiple dose combinations, a comparably short mean lysis time of 47 minutes was induced by a fractional combination consisting of 6% of the tPA monotherapy dose (0.2 μg/ml) and 40% of the M5 monotherapy dose (6 μg/ml) (Fig. 3, 5). Since this fractional combination induced the same effect as the maximum achievable with monotherapy by either activator, it was consistent with synergy, as previously observed with tPA and native proUK by some investigators [12, 13] but not by others [14]. Fibrinogenolysis was less with the combination than with monotherapy and could be further attenuated by the addition of C1-inhibitor. Although the C1-inhibitor inhibited lysis by tPA as shown (Fig. 3B), it maintained its synergistic effect, indicating that tPA was present at some excess.

A similar combination of mini-dose tPA (5% of the standard dose) and low dose proUK (50% standard hourly dose) was previously tested *in vivo* in a clinical study. In the multicenter PATENT trial, 101 patients with AMI were treated [29]. The first 10 patients were treated with a 10 mg bolus of tPA which turned out to be excessive, and the remainder with a 5 mg bolus; each bolus was followed by a 40 mg per hour infusion of proUK for 90 minutes. A TIMI 2–3 coronary patency rate of 77% and a TIMI 3 patency rate of 60% were obtained, with no reocclusions or ICH and only one death in the entire study. This patency rate was comparable to that of full dose, front-loaded tPA in GUSTO [30], the study which led to the approval of tPA for AMI [31].

The efficacy of a small amount of tPA seen in the present study as well as in the PATENT trial, when in combination with proUK, reflects tPA’s biological role to initiate fibrinolysis. In the event of a vascular occlusion, the tPA stored in the vessel wall is released locally, binds to the clot, and initiates its degradation. The high fibrin affinity of tPA, mediated by both its finger and kringle-2 domains [32], induces tight binding to a site on the D-domain of intact fibrin [33] adjacent to plasminogen bound to lysine α157 [34]. The resulting ternary complex with fibrin promotes tPA plasminogen activation by about 1,000-fold [35]. Fibrinolysis is thereby initiated very efficiently, requiring little tPA as seen in Fig. 3A&B. Adding more tPA did not change the lysis rate of the combination (Fig. 4) indicating that the tPA lytic effect was limited to this first step.

**Fig 7:**
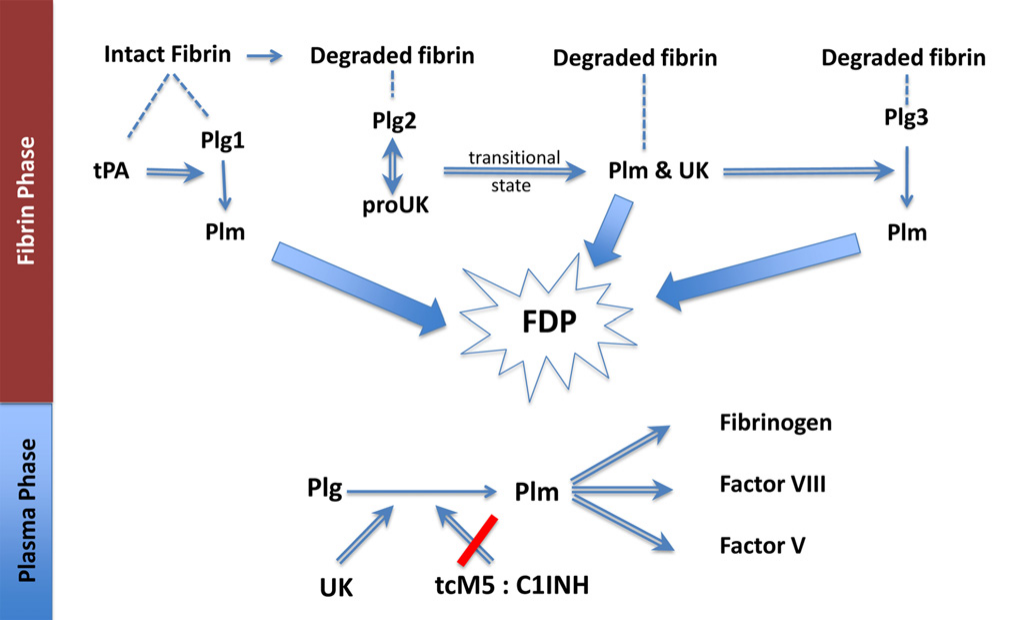
Fibrinolysis by the sequential modes of action of tPA and proUK or M5: tPA binds (dashed line) to intact fibrin adjacent to plasminogen(Plg1), forming a ternary complex and activation of Plg1 to plasmin (Plm). This initiates fibrin degradation resulting in the creation of new plasminogen binding sites on fibrin. Plg2 binds to a high-affinity binding site on the fibrin E (FFE) domain, which induces a special conformational shape change in Plg2 that enables it to be activated by the intrinsic activity of proUK. This proUK:plasminogen complex results in Plm formation and the reciprocal activation (double arrow) of proUK→urokinase (UK), associated with a transitional state that is hypercatalytic. UK, being a non-specific enzyme, then activates the remaining fibrin-bound plasminogen (Plg3). The three Plm together complete fibrin degradation (thick arrows) forming soluble fibrin degradation products (FDP). In the Plasma Phase, when UK diffuses off the fibrin clot, if it is not inhibited, it will activate plasma Plg to Plm, resulting in the degradation of 3 clotting factors, among other side effects. When M5 is used in place of proUK, this non-specific effect is prevented due to tcM5 being inhibited by C1INH, whose plasma concentration is >1,000-fold greater than that of the UK inhibitor.

ProUK/M5, by contrast, has no fibrin affinity and is a poor activator of plasminogen on intact fibrin. Instead proUK/M5 requires some degradation of the fibrin surface for its specific fibrinolytic effect [15]. As a result, there is a lag phase that precedes clot lysis by M5 (Fig. 3) or proUK [15], which can be overcome by a high dose (Fig. 1B), but at this dose, fibrin specificity is lost because of proUK conversion to the enzyme UK/tcM5 in plasma. After fibrin degradation is initiated by tPA, new plasminogen binding sites are created [36], including a high affinity site on the fibrin E-domain [37]. When plasminogen binds to this site, it undergoes a particular conformational change which makes it a favorable substrate for the proenzyme, proUK/M5, itself. Against this conformation, the intrinsic activity of proUK is promoted more than 250-fold, which gives it an activity equivalent to that of the enzyme, UK [38, 39], and a similar phenomenon was observed when solid phase fibrin underwent degradation [40]. After this plasminogen is activated by proUK; reciprocal activation of proUK by plasmin occurs [41] during which proUK goes through a transitional hypercatalytic stage [42]. After UK/tcM5 forms, being a non-specific plasminogen activator, it can then activate the remaining fibrin-bound plasminogen (Fig. 7).

During and after fibrinolysis, UK diffuses into the plasma where it activates plasma plasminogen which can induce side effects, since there is insufficient inhibitor in plasma for pharmacological UK concentrations. By contrast, tcM5 is effectively inhibited by C1-inhibitor which is relatively plentiful in plasma (~250 μg/ml). Being an acute phase reactant, it is up-regulated in disease states. C1-inhibitor is also available as a therapeutic agent. Since C1-inhibitor does not inhibit fibrinolysis by M5 (Fig. 2 and 3B), it can be used as protection against non-specific effects of fibrinolysis by M5 (Fig. 3B).

The contrasting properties of tPA and proUK/M5 account for their complementary effects in fibrinolysis and make their utilization in a sequential combination for thrombolysis more rational than using either activator alone. The present findings indicate that a small amount of tPA effectively initiates fibrinolysis which is then completed by proUK/M5 and UK/tcM5. ProUK is no longer available, but M5 is a safer alternative that is similarly synergistic with tPA as shown (Fig. 3A&B and Fig. 5).

The findings raise the question about the number of fibrin bound plasminogens available for fibrinolysis. This question was investigated in a study in which the molar amounts of plasminogen on intact and degraded fibrin were measured [43]. The investigators found 23.4 nmol/l on intact fibrin and 61.9 nmol/l on degraded fibrin. Since intact fibrin has only a single plasminogen binding site [33], these numbers are consistent with a total of three. Among these, tPA activates the first one [34, 35], whereas M5 and tcM5 activated the other two on degraded fibrin [38, 40] (Fig. 7). The same proUK:tPA 2:1 ratio implicated by this was found in a study in which the rates of fibrin-specific clot lysis (defined as <10% fibrinogen degradation) by tPA and by proUK in a plasma milieu were measured. The highest rate by proUK was consistently twice that by tPA [44], corresponding to the ratio of plasminogen on degraded and intact fibrin [43]. In the present study lysis by the M5 component in the synergistic combination was also almost twice (lysis time about half as long) that of the tPA component (Fig. 3A).

This additional fibrinolytic effect of proUK/M5 is a function of its activation to UK/tcM5 during fibrinolysis. When this was prevented by using an inactivatable proUK mutant, clot lysis was diminished by 100-fold [45]. By contrast to proUK/M5, tPA undergoes no functional change during fibrinolysis, since its single and two-chain forms have the same enzymatic and fibrinolytic properties [46], consistent with tPA’s more limited function in fibrinolysis.

In conclusion, clot lysis in a plasma milieu by a combination of mini-dose tPA and low-dose mutant proUK induced fibrinolysis of comparable efficacy to that by monotherapy at maximum doses of either activator, doses which are precluded by side effects in therapeutic thrombolysis with tPA [1]. The synergistic combination of tPA and M5 induced less fibrinogenolysis, which was further attenuated by supplemental C1-inhibitor. M5 was found to be synergistic with tPA as was proUK, and better suited for therapeutic use since it was more stable in plasma and its non-specific effects were inhibited by plasma C1-inhibitor (Fig. 7).
